# Human steroid sulfatase induces Wnt/β-catenin signaling and epithelial-mesenchymal transition by upregulating Twist1 and HIF-1α in human prostate and cervical cancer cells

**DOI:** 10.18632/oncotarget.18645

**Published:** 2017-06-27

**Authors:** Sangyun Shin, Hee-Jung Im, Yeo-Jung Kwon, Dong-Jin Ye, Hyoung-Seok Baek, Donghak Kim, Hyung-Kyoon Choi, Young-Jin Chun

**Affiliations:** ^1^ College of Pharmacy and Center for Metareceptome Research, Chung-Ang University, Seoul 06974, Republic of Korea; ^2^ Department of Biological Sciences, Konkuk University, Seoul 05029, Republic of Korea

**Keywords:** steroid sulfatase, epithelial-mesenchymal transition, Wnt/β–catenin pathway, HIF-1α, Twist1

## Abstract

Steroid sulfatase (STS) catalyzes the hydrolysis of estrone sulfate and dehydroepiandrosterone sulfate (DHEAS) to their unconjugated biologically active forms. Although STS is considered a therapeutic target for estrogen-dependent diseases, the cellular functions of STS remain unclear. We found that STS induces Wnt/β-catenin s Delete ignaling in PC-3 and HeLa cells. STS increases levels of β-catenin, phospho-β-catenin, and phospho-GSK3β. Enhanced translocation of β-catenin to the nucleus by STS might activate transcription of target genes such as cyclin D1, c-myc, and MMP-7. STS knockdown by siRNA resulted in downregulation of Wnt/β-catenin signaling. β-Catenin/TCF-mediated transcription was also enhanced by STS. STS induced an epithelial-mesenchymal transition (EMT) as it reduced the levels of E-cadherin, whereas levels of mesenchymal markers such as N-cadherin and vimentin were enhanced. We found that STS induced Twist1 expression through HIFα activation as HIF-1α knockdown significantly blocks the ability of STS to induce Twist1 transcription. Furthermore, DHEA, but not DHEAS is capable of inducing Twist1. Treatment with a STS inhibitor prevented STS-mediated Wnt/β-catenin signaling and Twist1 expression. Interestingly, cancer cell migration, invasion, and MMPs expression induced by STS were also inhibited by a STS inhibitor. Taken together, these results suggest that STS induces Wnt/β-catenin signaling and EMT by upregulating Twist1 and HIF-1α. The ability of STS to induce the Wnt/β-catenin signaling and EMT has profound implications on estrogen-mediated carcinogenesis in human cancer cells.

## INTRODUCTION

Steroid sulfatase (STS) hydrolyzes estrone sulfate (E1S) and dehydroepiandrosterone sulfate (DHEAS) to their unconjugated biologically active forms [1, 2], which modulate the growth and survival of estrogen-dependent cancers such as those of the prostate and breast [3]. Metabolic activation of E1S has been suggested as a major factor in carcinogenesis. Expression of STS is increased in breast cancer tissues and has prognostic importance [4]. Previous reports suggest that it is strongly recommended to monitor STS expression in estrogen-dependent neoplasia such as breast and endometrial cancers [5, 6]. Patients with high mRNA levels for STS are associated with an increased risk of breast cancer recurrence after surgery [6]. Because STS has a postulated significant role in the carcinogenicity of estrogens, STS is considered as a target enzyme for blocking estrogen-mediated carcinogenesis, and potent STS inhibitors have been developed and tested in rodents and in postmenopausal women with breast cancer [7]. STS expression has also been considered important for *in situ* androgen production as well as estrogen production in human prostate cancers [5].

Dehydroepiandrosterone (DHEA) is one of the major metabolites produced by STS from less active DHEAS. It acts predominantly as an endogenous precursor of more potent androgens such as testosterone and dihydrotestosterone in approximately 30-50% of circulating androgens in men and up to 100% of circulating estrogens in postmenopausal women [8]. Although DHEA has immunoregulatory functions and age-related DHEA alteration have been studied, the effect of DHEA on cancer cell growth is contradictory. DHEA may stimulate cancer growth in various types of cancer that are sensitive to steroids including breast, prostate, and uterine cancer. In addition, DHEA promotes benign prostatic hyperplasia in men. Moreover, DHEA as well as DHEAS are positively associated with breast cancer risk, particularly for ER positive/PR positive tumors [9]. When cells were exposed to physiological concentrations of DHEA (10^-8^ to 10^-9^ M), proliferation of MCF-7 cells was significant, but high concentrations of DHEA (10^-4^ to 10^-5^ M) strongly inhibits cell growth and induces autophagic cell death in HepG2 and HeLa cells [10, 11]. Therefore, detailed mechanisms of how STS expression and DHEA can induce proliferation in cancer cells are needed.

The Wnt/β-catenin signaling pathway includes a network of proteins well known for their roles in cancer [12–14]. When aberrantly activated, this signaling pathway leads to the accumulation of β-catenin in the cytoplasm, translocation of β-catenin to the nucleus to trigger the β-catenin/T-cell factor/lymphoid enhancer factor (TCF/LEF) transcriptional machinery, and upregulation of target genes, such as those encoding cyclin D1, c-myc, and matrix metalloproteinase (MMP)-7 [15]. Under normal conditions, β-catenin is degraded by a multi-protein degradation complex, and is maintained at low levels in the cytoplasm through continuous degradation by the 26S ubiquitin-proteasome pathway [16, 17]. The tumor suppressor protein Axin acts as the scaffold for this complex by directly interacting with adenomatous polyposis coli, glycogen synthase kinase 3β (GSK3β), casein kinase I (CKI), and β-catenin [18, 19]. This process is regulated by the Wnt/β-catenin signaling cascade, which inhibits GSK3β and thus β-catenin degradation [20, 21].

Several studies indicate that Wnt/β-catenin signaling plays a crucial role in epithelial–mesenchymal transition (EMT) [22–25]. Down-regulation of E-cadherin, which releases free β-catenin, correlates with EMT in colon epithelial cells [26–31]. Several up-regulated target genes of the Wnt/β-catenin signaling pathway such as fibronectin and MMP-7, correlate with a mesenchymal phenotype and invasiveness [32, 33]. In addition, estrogen enhances reversible EMT and collective motility in MCF-7 breast cancer cells [34–36]. Tumor cells with nuclear β-catenin accumulation appear to undergo EMT, as shown by the progressive loss of E-cadherin and the acquisition of mesenchymal markers such as vimentin and N-cadherin [12, 14, 35, 36]. EMT also plays an important role in cancer metastasis [14, 37]. Thus, Wnt/β-catenin signaling and EMT may act synergistically during carcinogenesis.

To study the functional role of STS on the Wnt/β-catenin signaling pathway and EMT to elucidate how STS expression modulates cancer progression in human cancer cells, we measured multiple hallmarks of cancer progression including cancer cell invasion and migration following STS overexpression or knockdown. Moreover, to determine the importance of STS-mediated steroid metabolism, the effects of DHEA and DHEAS on EMT were compared. We investigated further the interplay between STS, HIF-1α, and Twist1, which contributes to the gene expression responsible for EMT. We show that STS-induced Twist1 expression is mediated in a HIF-1α–dependent manner in human prostate and cervical cancer cells. These findings provide novel insight into how high level expression of STS in cancer can promote cancer progression and metastasis.

## RESULTS

### STS activates Wnt/β-catenin signaling

**Figure 1:**
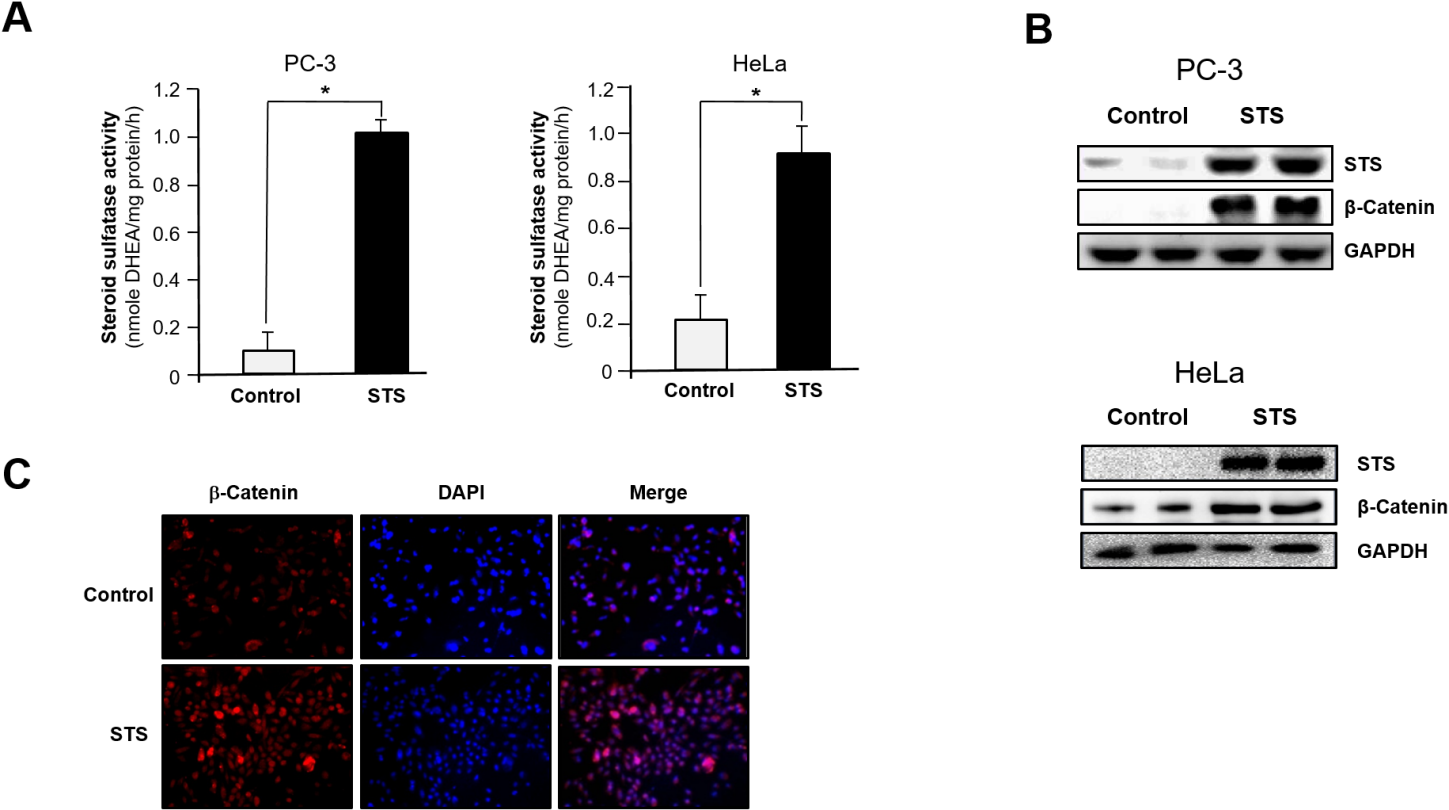
STS induces β-catenin expression in cancer cells Cells were transfected with pcDNA3.1-STS for 48 h. **(A)** STS activity. PC-3 and HeLa cell lysates were prepared and incubated with DHEAS (50 *μ*M). The metabolite generated was analyzed by using UPLC. Specific activity of STS was determined and each data point represents the mean ± S.E.M. of three experiments. *p<0.05 compared with control. **(B)** Total cellular lysates were prepared and used for Western blot analyses with antibodies against STS or β-catenin. GAPDH was used as a loading control. **(C)** Immunofluorescence staining. PC-3 cells were cultured on cover slips and transfected with pcDNA3.1-STS for 48 h. Cells were fixed and incubated with β-catenin antibody, stained with Alexa Fluor 594-labeled secondary antibody, and photographed by fluorescence microscopy.

To understand the role of STS in cancer progression, we studied the correlation between STS expression and Wnt/β-catenin signaling. When PC-3 cells were transfected with an STS expression vector (pcDNA3.1-STS), significantly greater STS activity was observed in STS-overexpressing cells than in control cells (1024 ± 51 pmole DHEA/mg protein/h vs. 93 ± 74 pmole DHEA/mg protein/h). In HeLa cells, STS activity was also induced by transfection of STS (913 ± 114 pmole DHEA/mg protein/h vs. 238 ± 96 pmole DHEA/mg protein/h) (Figure 1A). In addition, the level of STS protein was markedly increased by STS overexpression (Figure 1B). β-Catenin levels were also strongly increased in PC-3 cells overexpressing STS. Furthermore, β-catenin was increased by overexpressing STS in HeLa (Figure 1B). As shown in Figure 1C, STS expression was able to induce β-catenin expression, as determined by immunofluorescence analysis in PC-3 cells.

**Figure 2:**
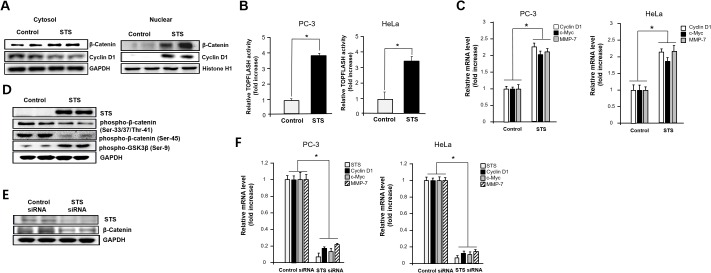
STS increases nuclear translocation of β-catenin and activation of downstream target genes **(A)** PC-3 cells were transfected with pcDNA3.1-STS for 48 h. After harvesting, the nuclear fraction was isolated, and total nuclear protein (20 *μ*g) was subjected to Western blot analysis with anti-β-catenin or cyclin D1 antibodies. Histone H1 was used as a nuclear marker and GAPDH was used as a cytosolic marker. **(B)** Cells were transfected with pcDNA3.1-STS, TOPFLASH, and renilla luciferase vectors for 24 h. Cells were subjected to the dual-luciferase assay. The relative firefly luciferase activity, normalized by the renilla luciferase activity, is shown. Each data point represents the mean ± S.E.M. of three experiments. *p<0.05 compared with control. **(C)** Cells were transfected with pcDNA3.1-STS for 48 h. Total RNA was isolated, and mRNA expression of cyclin D1, c-myc, and MMP-7 genes were determined by qPCR. **(D)** PC-3 cells were transfected with pcDNA3.1-STS for 48 h. Total cellular protein (20 *μ*g) was subjected to Western blot analysis with anti-phospho-GSK3β (Ser-9), phospho-β-catenin (Ser-33/37/Thr-41), or phospho-β-catenin (Ser-45) antibodies. **(E)** PC-3 cells were transfected with STS siRNA (50 nM) for 48 h. Total cellular proteins (20 *μ*g) were subjected to Western blot analysis with anti-STS or β-catenin antibodies. GAPDH was used as a loading control. **(F)** Cells were transfected with STS siRNA (50 nM) for 48 h. Total RNA was isolated and mRNA expression of STS, cyclin D1, c-myc, and MMP-7 were determined by qPCR.

Because cytosolic β-catenin accumulation induces its translocation into the nucleus, where it interacts with various transcription factors of the TCF family such as TCF4, we determined whether STS increases β-catenin/TCF4 transcriptional activity [17, 19, 38–40]. STS expression induced β-catenin accumulation in the nucleus, and the level of cyclin D1, a downstream target of β-catenin, was also strongly increased (Figure 2A). Overexpression of STS caused an approximately 4-fold increase of TOPFLASH activity in PC-3 and HeLa cells (Figure 2B). As the β-catenin/TCF complex activates the transcription of specific target genes [21, 41], we measured the mRNA levels of cyclin D1, c-myc, and MMP-7 genes after STS overexpression in both PC-3 and HeLa cells. As shown in Figure 2C, STS was able to induce expression of all of these genes. Previous report showed that GSK3β and CKIα may control β-catenin level by phosphorylation of β-catenin on Ser-33/37/Thr-41 and Ser-45, respectively, which can promote ubiquitin-dependent degradation of β-catenin [42]. In contrast, phosphorylation of GSK3β on Ser-9 by various serine/threonine kinases including PKA, PKC, Akt, p70^S6K^, or p90^RSK^ which inhibits GSK3 kinase activity can enhance the stability of cytoplasmic β-catenin [43, 44]. As expected, STS decreased β-catenin phosphorylation on Ser-33/37/Thr-41 and on Ser-45, but it induced the phosphorylation of GSK3β on Ser-9 (Figure 2D). To ensure the role of STS on β-catenin expression, the effect of STS gene knockdown was determined. When cells were treated with STS siRNA, both protein and mRNA levels of STS were reduced (Figure 2E and 2F). STS siRNA also significantly suppressed the protein level of β-catenin (Figure 2E) and the mRNA levels of cyclin D1, c-myc, and MMP-7 (Figure 2F).

### STS represses E-cadherin and induces N-cadherin and vimentin

**Figure 3:**
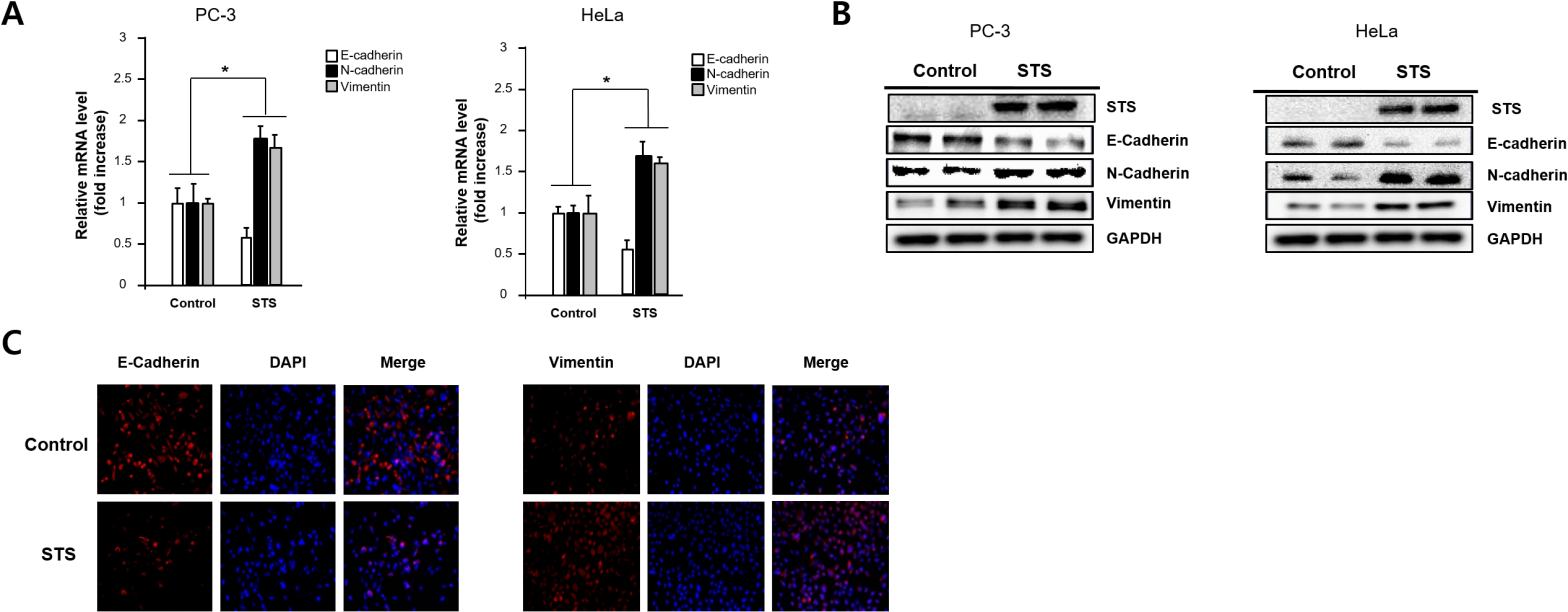
STS induces EMT in both PC-3 and HeLa cells Cells were transfected with pcDNA3.1-STS for 48 h. **(A)** Total RNA was isolated and mRNA expression of STS, E-cadherin, and N-cadherin were determined by qPCR. **(B)** Total cellular protein (20 *μ*g) was subjected to Western blot analysis with STS, E-cadherin, N-cadherin, and vimentin antibodies. GAPDH was used as a loading control. **(C)** PC-3 cells were cultured on cover slips and transfected with pcDNA3.1-STS for 48 h. Cells were fixed, incubated with E-cadherin or vimentin antibody, stained with Alexa Fluor 594-labeled secondary antibody, and photographed by fluorescence microscope.

To test whether STS expression is able to induce EMT, the levels of E-cadherin, N-cadherin, and vimentin were measured in PC-3 and HeLa cells. Quantitative real-time PCR analysis showed E-cadherin suppression and induction of N-cadherin and vimentin in STS-overexpressing PC-3 and HeLa cells compared with control cells (Figure 3A). Western blot analysis also showed E-cadherin repression, which is coincided with induction of N-cadherin and vimentin (Figure 3B). Immunofluorescence analysis of E-cadherin and vimentin confirmed that STS might induce EMT in PC-3 cells (Figure 3C).

### STS induces Twist1 expression through hypoxia response element

**Figure 4:**
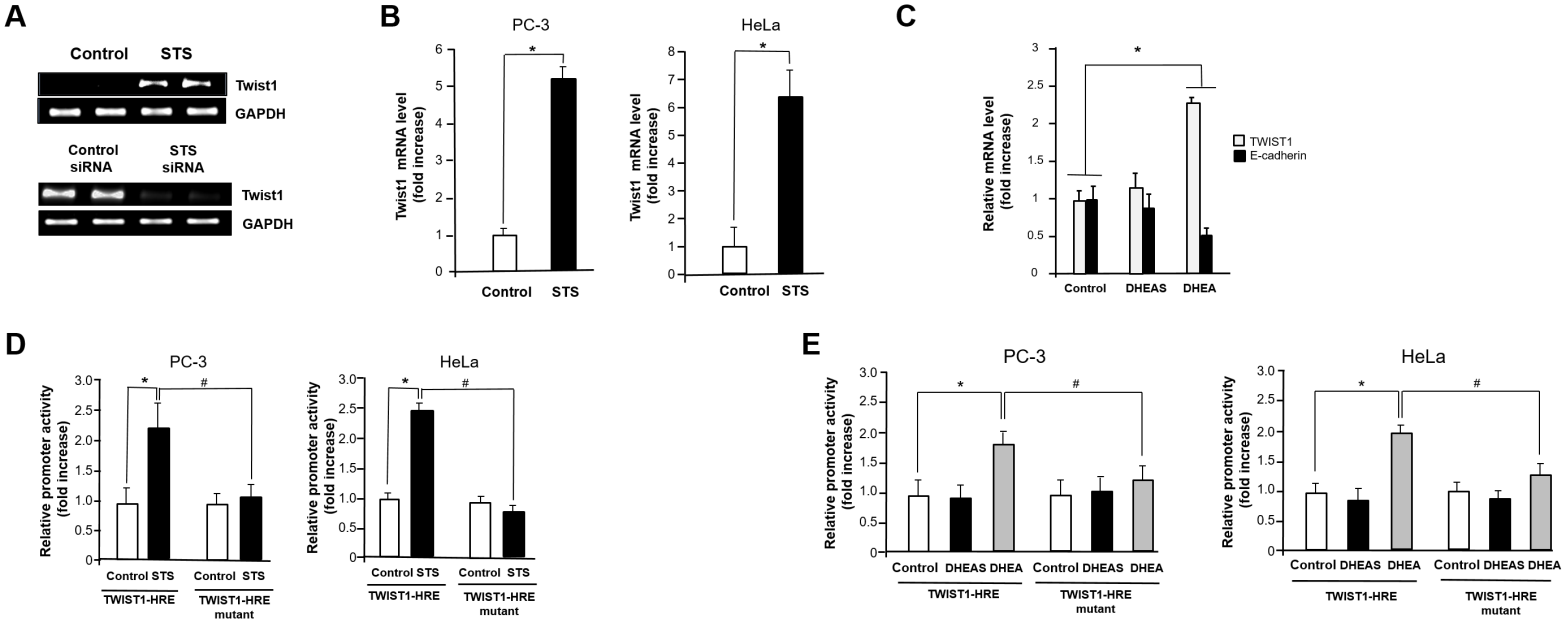
STS induces Twist1 expression in cancer cells **(A)** PC-3 cells were transfected with pcDNA3.1-STS or STS siRNA (50 nM) for 48 h. Total RNA was isolated, and mRNA expression of Twist1 was determined by qPCR. Expression of GAPDH mRNA was determined as an RNA control. **(B)** Cells were transfected with pcDNA3.1-STS. Total RNA was isolated, and mRNA expression of Twist1 was determined by qPCR. Each data point represents the mean ± S.E.M. of three experiments. *p<0.05 compared with control. **(C)** Cells were treated with DHEA or DHEAS (100 nM) for 24 h. Total RNA was isolated, and mRNA expression of Twist1 and E-cadherin were determined by qPCR. Expression of GAPDH mRNA was determined as an RNA control. **(D)** Cells were transfected with pcDNA3.1-STS, Twist1-HRE promoter, Twist1-HRE mutant promoter, and renilla luciferase vector for 24 h. The relative firefly luciferase activity, normalized by the renilla luciferase activity, is shown. Each data point represents the mean ± S.E.M. of three experiments. *p<0.05 compared with control. ^#^*p*<0.05 compared with Twist1-HRE promoter vector transfected cells. **(E)** Cells were transfected with Twist1-HRE promoter, Twist1-HRE mutant promoter, and renilla luciferase vector for 24 h and then were treated with DHEA or DHEAS (100 nM) for 24 h. The relative firefly luciferase activity, normalized by the renilla luciferase activity, is shown. Each data point represents the mean ± S.E.M. of three experiments. *p<0.05 compared with control. ^#^*p*<0.05 compared with Twist1-HRE promoter vector transfected cells.

Because Twist1 is known to induce EMT in human mammary cells and can act as one of the main regulators of EMT [45, 46], we examined the mRNA expression of Twist1 in response to STS expression to determine if STS activates EMT through induction of Twist1 gene expression. We found that STS induced mRNA expression of Twist1. However, STS siRNA almost completely abrogated expression of Twist1 (Figure 4A). STS markedly increased mRNA levels of Twist1 in both PC-3 and HeLa cells (Figure 4B). DHEA also induced Twist1, and it suppressed E-cadherin expression in PC-3 cells although DHEAS failed to increase the expression of Twist1 (Figure 4C). These results strongly indicate that STS may promote EMT through activating Twist1 expression.

To determine how STS induces Twist1 expression, we tested the ability of STS to activate the Twist1 promoter by luciferase reporter assay. When cells were transfected with the Twist1-HRE reporter (pTWIST1-120), about 2- and 2.5-fold induction of Twist1 promoter activity were observed in PC-3 and HeLa cells expressing STS, respectively. Interestingly, transfection with a Twist1-HRE mutant (pTWIST1-120/HRE-mut) containing a mutant HRE site (^-83^ACAGT^-79^) completely abolished promoter activity in both PC-3 and HeLa cells, indicating a HRE site (^-83^CACGT^-79^) may be crucial for Twist1 induction by STS (Figure 4D). As shown in Figure 4E, we found that DHEA was able to induce Twist1 promoter activity about 1.7-fold compared to the control in both cells. However, Twist1 promoter activities increased by STS expression was abrogated when cells were transfected with Twist1-HRE mutant. Again, DHEAS had no significant effects in both cells transfected with Twist1-HRE or Twist1-HRE mutant (Figure 4E). These results suggest that STS or its metabolite DHEA may control the HRE site on the human Twist1 promoter to activate its transcription.

### Twist1 induction by STS is HIF-1α dependent

**Figure 5:**
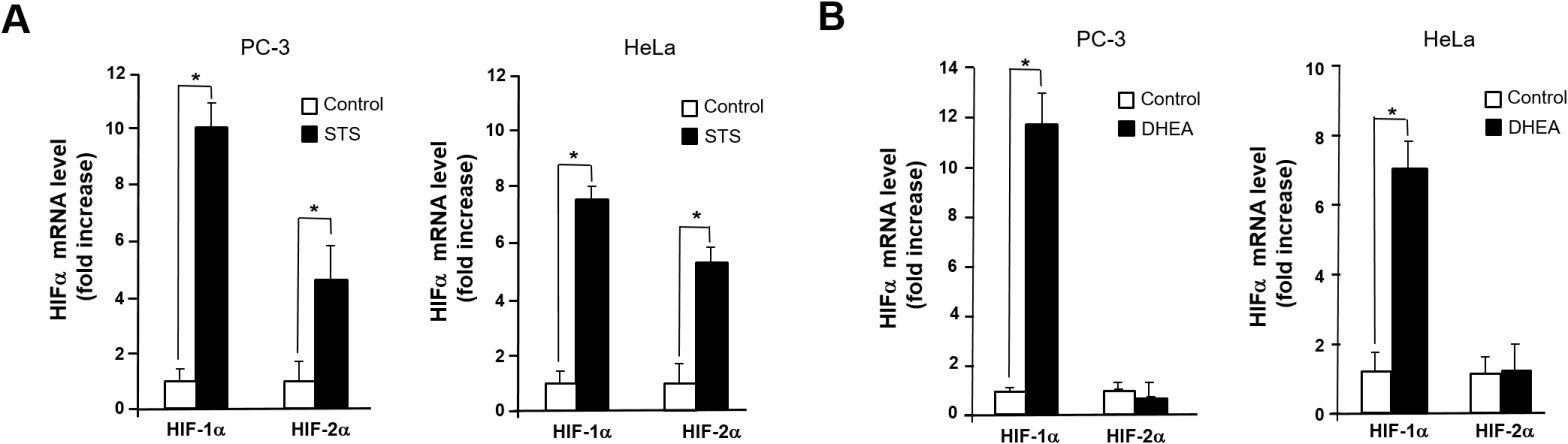
STS induces transcription of HIF-1α and HIF-2α **(A)** Cells were transfected with pcDNA3.1-STS. Total RNA was isolated, HIF-1α and HIF-2α genes were amplified with specific primers. **(B)** Cells were treated with DHEA (100 nM). Total RNA was isolated, mRNA expression of HIF-1α and HIF-2α were determined by qPCR. Genes were amplified with specific primers. Each data point represents the mean ± S.E.M. of three experiments. *p<0.05 compared with control.

**Figure 6:**
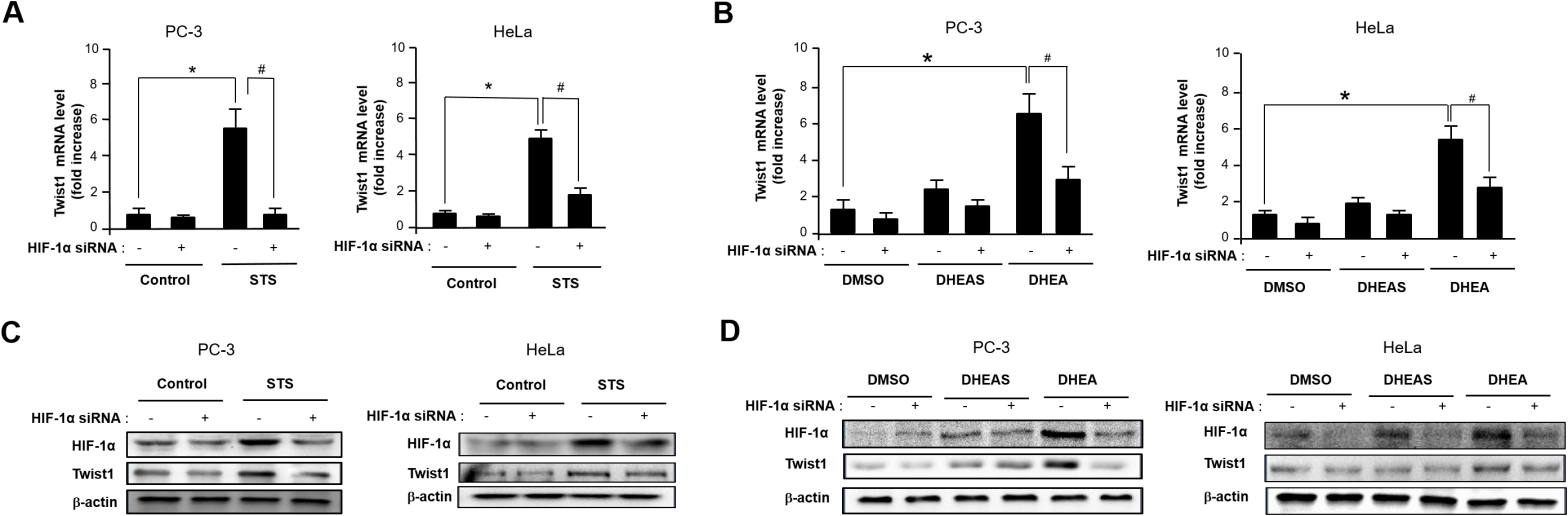
STS and DHEA enhance Twist1 expression through HIF-1α induction **(A)** Cells were transfected with pcDNA3.1-STS and HIF-1α siRNA for 48 h. Total RNA was isolated and mRNA expression of Twist1 was determined by qPCR. Each data point represents the mean ± S.E.M. of three experiments. *p<0.05 compared with control. ^#^*p*<0.05 compared with STS-overexpressed cells. **(B)** Cells were treated with DHEA (100 nM), or DHEAS (100 nM), and transfected with HIF-1α siRNA for 48 h. mRNA expression of Twist1 was determined by qPCR. Each data point represents the mean ± S.E.M. of three experiments. *p<0.05 compared with control. ^#^*p*<0.05 compared with STS-overexpressed cells. **(C)** Cells were transfected with pcDNA3.1-STS and HIF-1α siRNA for 48 h. Total cellular protein (20 μg) was subjected to Western blot analysis with HIF-1α and Twist1 antibodies. β-Actin was used as a loading control. **(D)** Cells were treated with DHEA (100 nM) or DHEAS (100 nM), and transfected with HIF-1α siRNA for 48 h. Total cellular protein (20 μg) was subjected to Western blot analysis with HIF-1α and Twist1 antibodies. β-Actin was used as a loading control.

Given that activation of HRE site is required for inducing Twist1 expression by STS, we tested whether HIF-1α is essential for STS-mediated Twist1 expression. As shown in Figure 5A, HIF-1α and HIF-2α mRNA transcription was significantly induced by STS expression in PC-3 and HeLa cells. Interestingly, DHEA was able to induce HIF-1α transcription but it failed to induce HIF-2α transcription (Figure 5B). STS-mediated Twist1 induction was HIF-1α-dependent because HIF-1α siRNA strongly attenuated Twist1 expression by STS in mRNA and protein levels (Figure 6A and 6C). In addition, HIF-1α siRNA also repressed DHEA-mediated Twist1 induction (Figure 6B and 6D). These results demonstrate that STS and DHEA may induce Twist1 expression via HIF-1α activation.

### Prevention of STS-induced EMT by specific STS inhibitor

**Figure 7:**
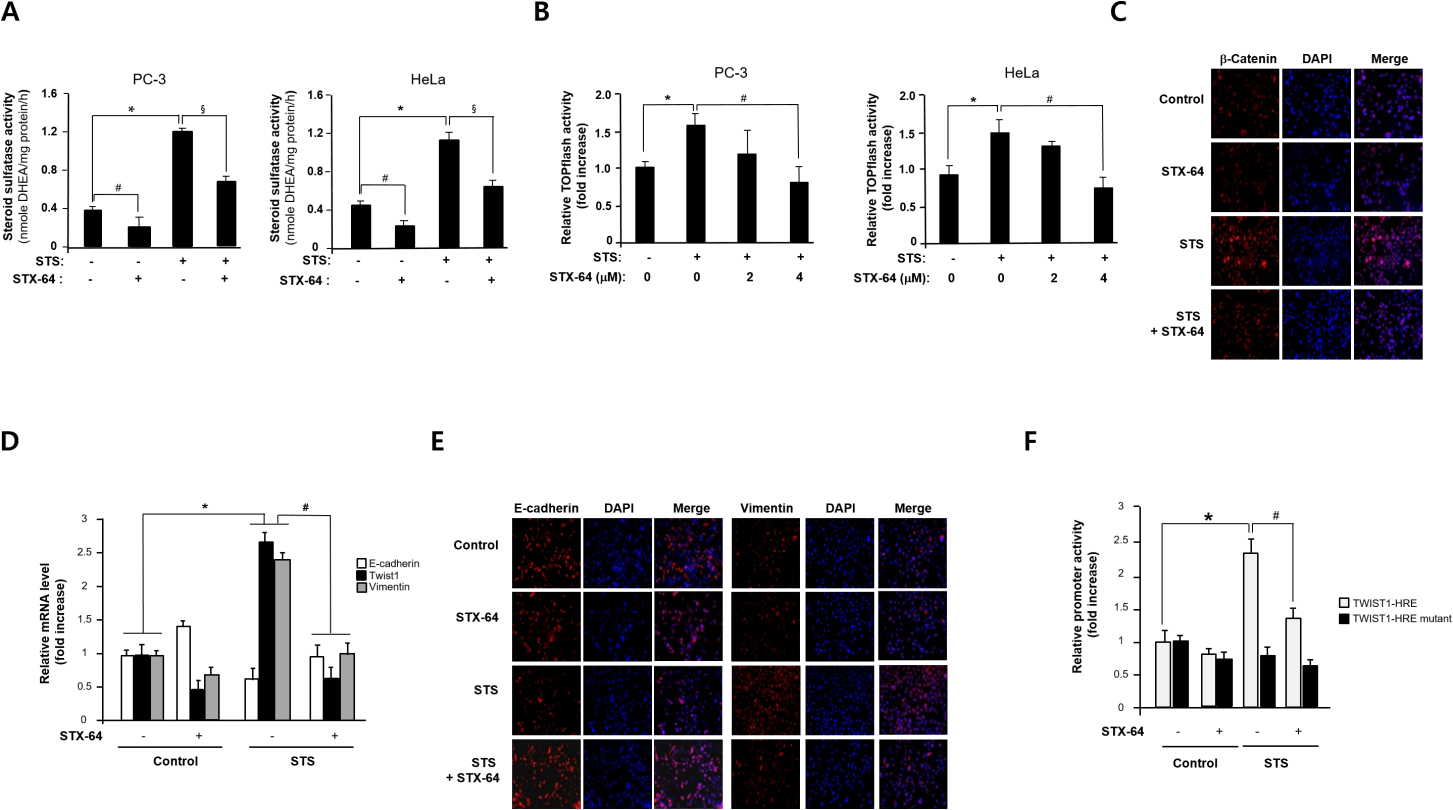
STX-64 suppresses STS-mediated β-catenin expression **(A)** STS activity. Cells were transfected with pcDNA3.1-STS and treated with STX-64 (2 μM) for 24 h. Cell lysates were prepared and incubated with DHEAS (50 μM). The metabolite generated was analyzed by using UPLC. Specific activity of STS was determined and each data point represents the mean ± S.E.M. of three experiments. *p<0.05 compared with control. ^#^*p*<0.05 compared with DHEA-treated cells. ^§^*p*<0.05 compared with only STS-overexpressed cells. **(B)** Cells were transfected with pcDNA3.1-STS, TOPFLASH, and renilla luciferase vector for 24 h in the presence or absence of STX-64. Cells were subjected to the dual-luciferase assay. The relative firefly luciferase activity, normalized by the renilla luciferase activity, is shown. ^*^p<0.05 compared with control. ^#^*p*<0.05 compared with only STS-overexpressed cells. **(C)** Immunofluorescence staining. PC-3 cells were transfected with pcDNA3.1-STS and treated with STX-64 (4 *μ*M) for 48 h. Cells were incubated with β-catenin antibody, stained with Alexa Fluor 594-labeled secondary antibody, and photographed by fluorescence microscopy. **(D)** PC-3 cells were transfected with pcDNA3.1-STS and treated with STX-64 (2 *μ*M) for 24 h. Total RNA was isolated and mRNA expression of Twist1, E-cadherin, and vimentin were determined by qPCR. ^*^p<0.05 compared with untreated control cells. ^#^*p*<0.05 compared with only STS-overexpressed cells. **(E)** Immunofluorescence staining. PC-3 cells were transfected with pcDNA3.1-STS and treated with STX-64 (4 *μ*M) for 48 h. Cells were incubated with E-cadherin or vimentin antibody, stained with Alexa Fluor 594-labeled secondary antibody, and photographed by fluorescence microscopy. **(F)** Cells were transfected with pcDNA3.1-STS, Twist1-HRE promoter, Twist1-HRE mutant promoter, and renilla luciferase vector and treated with STX-64 (2 μM) for 24 h. The relative firefly luciferase activity, normalized by the renilla luciferase activity, is shown. ^*^p<0.05 compared with untreated control cells. ^#^*p*<0.05 compared with only STS-overexpressed cells.

To determine whether STS enzyme activity is important for EMT induction, cells were treated with STX-64, a potent and irreversible inhibitor of STS [47, 48], STS enzyme activity in overexpressing cells was significantly abolished by treating with STX-64 (2 μM) in PC-3 and HeLa cells (Figure 7A). Moreover, STX-64 was able to inhibit TOPFLASH activity induced by STS (Figure 7B). We conducted immunofluorescence analysis to see whether STX-64 inhibits STS-mediated β-catenin expression and found similar effects (Figure 7C). We confirmed repression of E-cadherin and an increase in mRNA levels of Twist1 and vimentin in the STS-transfected cells as previously reported (Figure 7D). Inhibition of STS by STX-64 increased E-cadherin expression whereas it repressed Twist1 and vimentin mRNA expression (Figure 7D). Immunofluorescence analysis of E-cadherin and vimentin confirmed that STS regulates E-cadherin and vimentin expression, while these effects were inhibited by STX-64 (Figure 7E).

Twist1 promoter activities induced by STS was significantly decreased by STX-64. When PC-3 cells were transfected with Twist1-HRE mutant, no significant changes were shown in STS-overexpressing cells and STX-64-treated cells (Figure 7F). These results indicate that STS activity may be important for activating Twist1 mRNA transcription through HRE site(s).

### Suppression of cancer cell migration and invasion by STS inhibitor

**Figure 8:**
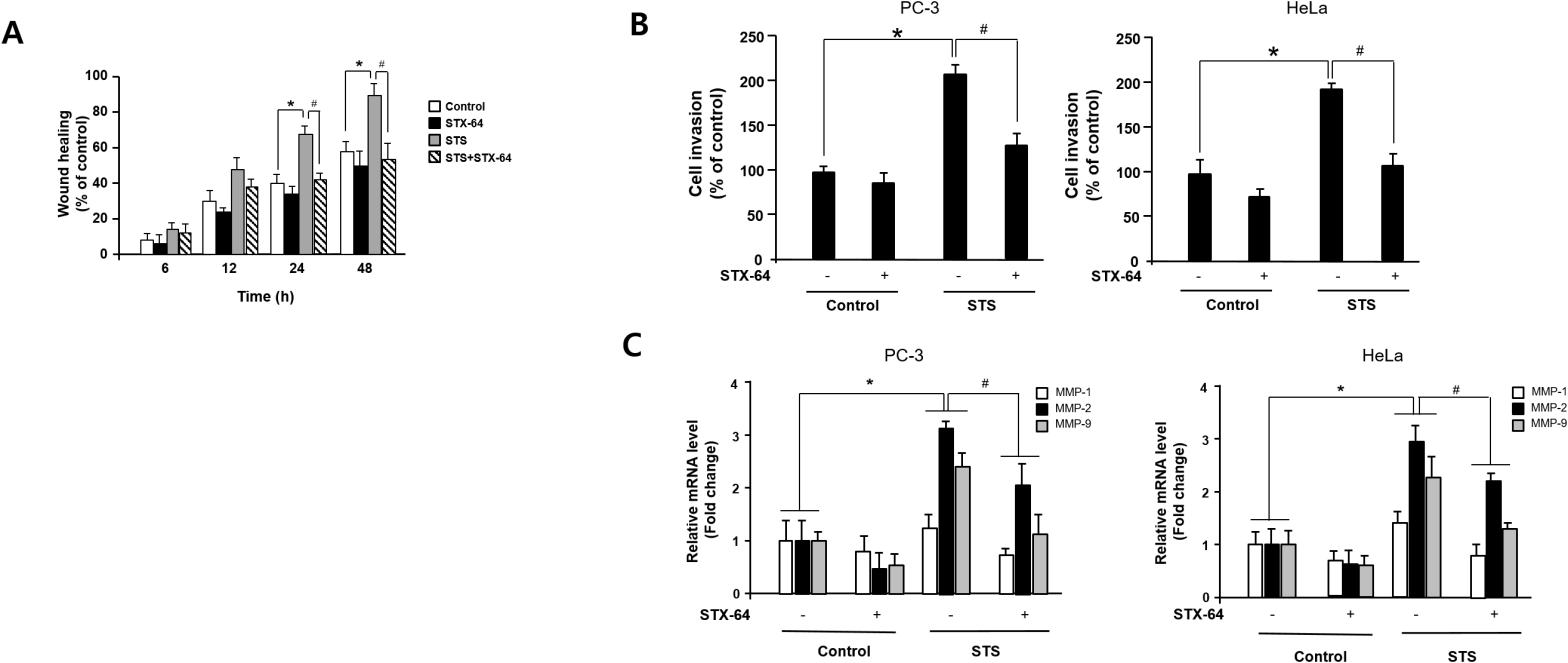
STS enhances cell migration and invasion in human cancer cells **(A)** Scratch wound assay. Cells were transfected with pcDNA3.1-STS, and treated with STX-64 (4 *μ*M) for 24 h. An injury line was created, and plates were photographed at 0 h to 48 h. **(B)** Invasion assay. Cells were transfected with pcDNA3.1-STS for 24 h and transferred to an invasion chamber insert. Cell invasion was analyzed by using QCM^TM^ 24-well Cell Invasion Assay Kits. ^*^p<0.05 compared with untreated control cells. ^#^*p*<0.05 compared with only STS-overexpressed cells. **(C)** qPCR. Cells were transfected with pcDNA3.1-STS, and treated with STX-64 (4 *μ*M) for 24 h. Total RNA was isolated and mRNA expression of MMP-1, MMP-2, and MMP-9 were determined by qPCR. Each data point represents the mean ± S.E.M. of three experiments. ^*^p<0.05 compared with untreated control cells. ^#^*p*<0.05 compared with only STS-overexpressed cells.

Because Twist1 is known to be a master regulator of cell migration and metastasis and STS is capable of inducing Twist1 expression [46], the effect of STS on tumor cell motility was investigated using the scratch wound assay and the Boyden chamber invasion assay. STS overexpression resulted in a twofold enhancement of migration of PC-3 cells into the scratched area (Figure 8A). STX-64 (4 *μ*M) significantly inhibited STS-mediated cell motility. We also found that STS expression in both HeLa and PC-3 cells rendered them to become highly invasive and STX-64 was able to prevent STS-mediated cancer cell invasion (Figure 8B). To determine whether MMPs play an important role in STS-mediated cancer cell invasion, we investigated the expression of MMP-1, MMP-2, and MMP-9 mRNA in PC-3 or HeLa cells expressing STS after exposure to STX-64. MMP-2 and MMP-9 mRNA levels were strongly enhanced by STS and STX-64 caused a significant repression of MMPs mRNA expression induced by STS (Figure 8C). These data demonstrated that STS activity may promote cancer cell migration and invasion through Twist1 and MMP induction.

## DISCUSSION

STS has a crucial role in regulating estrogen biosynthesis within hormone-dependent cancers such as prostate and breast cancers. STS is involved in local production and metabolism of estrogens in human prostate cancers [5]. The STS mRNA expression in malignant breast tissue seems to be significantly higher than in normal tissue [6]. A strong correlation between high levels of STS mRNA and poor survival was also suggested [40]. Moreover, STS-dependent pathway may play an important role in tumor growth and survival.

In this study, we demonstrated how STS affects tumor progression and metastasis. Our data clearly showed that STS overexpression induces the Wnt/β-catenin signaling and EMT in PC-3 cells as well as HeLa cells. We observed that STS overexpression significantly increased β-catenin expression and translocation of β-catenin to the nucleus. Nuclear β-catenin increases the TCF reporter activity and enhances β-catenin target gene expression such as cyclin D1, c-myc, and MMP-7. The role of STS in inducing the β-catenin-mediated pathway was also confirmed by STS knockdown. Our results indicate that E-cadherin level was downregulated by STS. An inverse correlation between β-catenin and E-cadherin has been extensively studied. E-cadherin interferes nuclear translocation and transcriptional activity of β-catenin by forming the complex at the membrane [41]. However, β-catenin might negatively regulate E-cadherin expression by activating E-cadherin repressors such as ZEB1, ZEB2, Snail, and Slug [49]. Twist1, which is known to be a potential target of β-catenin also can suppress E-cadherin level through the E-boxes on the E-cadherin promoter [33, 50, 51].

We showed that STS may stabilize cytosolic β-catenin by suppressing phosphorylation of β-catenin because phosphorylation of β-catenin on Ser-33/37/Thr-41 by GSK3β and on Ser-45 by CKIα was decreased when STS was overexpressed. Our results also showed that STS increased phosphorylation of GSK3β on Ser-9, which may enhance ubiquitin-dependent degradation of GSK3β. Previous studies have reported that DHEA and its metabolites significantly increase β-catenin expression and TCF reporter activity via increasing Gαq/Dishvelled2 (Dvl2) association in DU145 cells. Overexpression of Gαq in PC-3 cells constitutively activates β-catenin target gene expression through alterations of Gαq/Dvl2 association and reduction of GSK3-associated phosphorylation of β-catenin [43]. Because polymerized Dvl2 by activated LRP6 coreceptor is capable of interaction with Axin and recruit Axin together with GSK3 to LRP6 in the destruction complex, GSK-mediated phosphorylation of β-catenin is prevented as phosphorylated LRP6 binds to the catalytic site of GSK3, thereby blocking its activity towards β-catenin [44]. We found that STS induced LRP6 mRNA expression whereas STS siRNA suppressed its expression (data not shown). These results suggest that STS may induce β-catenin signaling through Gαq/LRP6/Dvl2 association although the involvement of Gαq in STS-mediated signaling need to be verified.

As mentioned above, Twist1 activation is required for STS-mediated cancer cell progression. Twist1 expression was strongly induced in STS-overexpressing cells. We also confirmed the inverse correlation between Twist1 and E-cadherin. Twist1 is a basic helix-loop-helix transcription factor known as a master regulator of EMT [33, 35]. It plays an important role in cancer progression and metastasis through various cellular processes including angiogenesis, extravasation, and invadopodia. Twist1 is overexpressed in various types of human cancers including breast, stomach, and prostate cancers, and is a prognostic marker of metastatic prostate cancer [52]. Induction of EMT by Twist1 is mainly caused by down-regulation of E-cadherin expression because Twist1 binds to the E-boxes of the CDH1 promoter and inhibits E-cadherin gene expression. Twist expression also has a close association with Akt signaling. Twist1 can induce Akt2 expression mainly through interaction with the E-box on its promoter [45]. Because activation of Akt2 by Twist1 results in the phosphorylation of the serine at residue 9 in GSK3β, which is followed by ubiquitination and degradation [46], induction of Twist1 by STS may promote Akt activation and then increases β-catenin stabilization and nuclear translocation.

Our data clearly showed that STS-induced Twist1 expression is mediated through HIF-1α expression because STS increased normal Twist promoter activity but had no effect on Twist HRE mutant promoter activity. In addition, we have also shown that DHEA enhances HIF-1α mRNA, protein, and promoter activity levels. These effects were almost prevented by HIF-1α siRNA. Previous study showed that direct binding of HIF-1α to HRE site located -83 to -79 upstream of the transcription start site of *TWIST*1 gene upregulates Twist1 expression in various cancer cells [53]. Mutation of the this HRE site prevented Twist1 promoter activity under HIF-1α overexpression or hypoxic condition. In our study, we used the same HRE site of Twist1 promoter and found that STS overexpression or DHEA treatment enhance promoter activity of Twist1 that is dependent on HRE site. Because Twist1 is a downstream target of HIF-1α, induction of HIF-1α by STS and DHEA may be one of the main pathways for STS-mediated EMT [54].

Although HIF-1α primarily contributes to promoting EMT and tumor progression, the role of HIF-2α needs to be considered as HIF-2α exhibits a distinct role in hypoxic expression and activation of target genes [55]. Our results showed that STS significantly induces HIF-2α mRNA transcription as well as HIF-1α. However, DHEA is able to induce HIF-1α but not HIF-2α transcription. This observation raises the question that there is some different mechanism between STS and DHEA to activate Twist1 expression. Gort et al. suggested that Twist1 gene is a direct target of HIF-2α because Twist1 expression in human cancer cells is enhanced by hypoxia in a HIF-2α-dependent manner [56]. However, in our case, when DHEA was tested for HIF-α and Twist1 expression, DHEA strongly induced Twist1 mRNA expression although DHEA did not show any significant effect on HIF-2α level. All these results lead to the idea that STS as well as DHEA may induce Twist1 expression mainly via HIF-1α activation.

To understand whether STS activity may be necessary for inducing EMT, the effects of a specific STS inhibitor STX-64 were determined. STX-64 is a tricyclic coumarin sulfamate inhibiting STS activity irreversibly with an IC_50_ value of 8 nM in placental microsomes and 1.5 nM in JEG-3 human choriocarcinoma cells [48, 57]. Treatment with STX-64 significantly inhibits metabolic activation of DHEAS to produce DHEA by STS and it can prevent TOPFLASH activity enhanced by STS expression. Moreover, induction of β-catenin and vimentin levels by STS was strongly decreased by STX-64, whereas repressed E-cadherin level by STS was recovered by STX-64. Inhibition of Twist1 mRNA expression by STX-64 may be mediated by suppressing HIF-1α activation because STX-64 is capable of preventing Twist1 promoter activity. As we know, these studies were the first to show that an inhibitor of STS can prevent Wnt/β-catenin signaling and EMT in cancer cells.

Our data from the scratch wound assay and invasion assay showed that STX-64 represses STS-mediated PC-3 and HeLa cell migration and invasion. Based on our results that STS induces MMPs mRNA expression such as MMP-1, 2, and 9 and STX-64 suppresses transcription of these MMPs, we suggest that those MMPs may play crucial roles in cell migration and invasion by STS. Recently we showed that STS and DHEA induce integrin β1 expression and phosphorylation of focal adhesion kinase, which serves as a mediator between integrin and MAPK/ERK pathway [58]. Previous studies have documented that integrin α5β1 confers invasiveness to cancer cells by regulating the MMP-2 activity and integrin β1 signaling is capable of suppressing RhoA activity and activating MMP-2-dependent cell migration [59, 60]. Because Twist1 transcriptionally activates integrin β1 expression [61], the possibility that induction of Twist1 by STS may activate MMP-2 expression for cell migration and invasion through integrin β1 signaling will need to be determined to understand the detailed mechanism of STS-mediated cancer progression.

**Figure 9:**
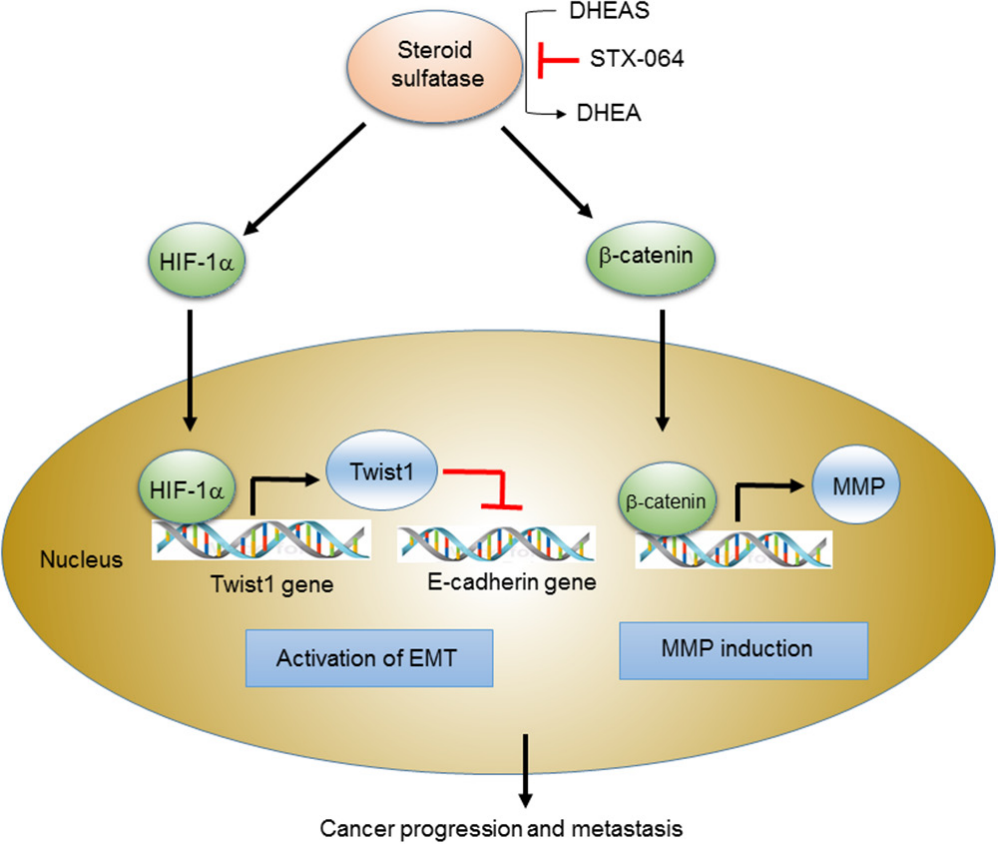
Scheme for the STS-mediated activation of Wnt/β-catenin signaling and EMT Scheme for STS-mediated cancer progression and metastasis through activation of Wnt/β-catenin signaling and EMT. STS overexpression and DHEA production trigger EMT program through HIF-1α-mediated Twist 1 expression as well as Wnt/β-catenin signaling by increasing cytosolic β-catenin level and nuclear translocation. They induce down-regulation of E-cadherin and up-regulation of MMP, promoting cancer progression and metastasis.

In summary, our present study suggests an important function of STS in human cancer cells. Our results imply that STS expression plays a significant role in activation of the Wnt/β-catenin signaling and the EMT. The scheme in Figure 9 summarizes the new findings that reveal the mechanism of STS-mediated carcinogenesis. The ability of STS to induce the Wnt/β-catenin pathway and the EMT program may cause a strong signal to cancer cell growth and has profound implications on estrogen-mediated carcinogenesis. Future studies are required to clarify the specific molecular mechanisms underlying the role of STS in cancer progression and metastasis in human cancer cells.

## MATERIALS AND METHODS

### Chemicals

A rabbit polyclonal antibody for STS was purchased from Abcam (Cambridge, UK). Antibodies against cyclin D1, β-catenin, vimentin, and GAPDH were purchased from Santa Cruz Biotechnology (Santa Cruz, CA). Antibodies against GSK3β, phospho-β-catenin (Ser-33/37/Thr-41), and phospho-β-catenin (Ser-45) were purchased from Cell Signaling Technology (Beverly, MA). Anti-phospho-GSK3β (Ser-9), N-cadherin, histone H1antibodies, and QCM^TM^ 24-well Fluorometric Cell Invasion Assay kit were obtained from Millipore (Bedford, MA). Anti-E-cadherin antibody was from Upstate Biotechnology (Lake Placid, NY). Dual-Luciferase Reporter Assay kit was purchased from Promega (Madison, WI). Ex-*Taq* DNA polymerase was obtained from TaKaRa Bio (Shiga, Japan). Other chemicals and reagents were of the highest quality commercially available.

### Cell culture

Human prostate cancer PC-3 cells and human cervical cancer HeLa cells were obtained from the Korean Cell Line Bank (Seoul, Korea). PC-3 cells were grown in RPMI 1640 medium supplemented with 10% heat-inactivated FBS, 100 U/ml penicillin, and 100 *μ*g/ml streptomycin. HeLa cells were grown in MEM medium supplemented with 10% heat-inactivated FBS, 100 U/ml penicillin, and 100 *μ*g/ml streptomycin. Cells were maintained at 37°C in a humidified atmosphere of 5% CO_2_. For treatment of cells with DHEA or DHEAS, 1×10^6^ cells were seeded in growth media as a monolayer in 100-mm plates. After 24 h, the media was changed to phenol red-free RPMI with 10% (v/v) charcoal-stripped FBS (Thermo Fisher), 100 U/ml penicillin, and 100 *μ*g/ml streptomycin. Cells were maintained for 48 h and were subsequently cultured in fresh media containing the designated concentrations of DHEA or DHEAS. After 48 h, cells were harvested and processed for further studies.

### Quantitative RT-PCR

Total RNA was extracted using Ribospin^TM^ (GeneALL, Seoul, Korea). Total RNA (500 ng) was transcribed at 37°C for 1 h in a volume of 20 *μ*l containing 5× RT buffer, 10 mM dNTPs, 40 units of RNase inhibitor, 200 units of Moloney murine leukemia virus reverse transcriptase, and 100 pmole of oligo(dT) primer. Subsequently, 0.8 *μ*l of the reaction mixture from each sample was amplified with 10 pmole of each oligonucleotide primer, 0.2 mM dNTPs, 1.5 mM MgCl_2_, and 1.25 units of Ex Taq DNA polymerase in a final volume of 25 *μ*l. PCR was performed as follows: one cycle of 95°C for 2 min, followed by 35 cycles of denaturation at 95°C for 10 s, annealing at 58°C for 15 s, and extension at 72°C for 15 s. The number of amplification cycles was optimized in preliminary experiments to ensure that the PCR amplification did not reach a plateau. PCR products were subjected to 2% (w/v) agarose gel electrophoresis and analyzed using a ChemiDoc XRS (Bio-Rad, Hercules, CA). Quantitative PCR (qPCR) was performed using the Rotor-Gene SYBR^®^ PCR Kit (Qiagen, Hilden, Germany), as recommended by the manufacturer. Each reaction contained 12.5 *μ*l of 2× SYBR^®^ Green PCR Master Mix, 10 pmole of each oligonucleotide primer, and 2 *μ*l of cDNA in a final volume of 25 *μ*l. Amplification was conducted as follows: one cycle at 95°C for 5 min, followed by 40 cycles of denaturation at 95°C for 5 s, and annealing/extension at 60°C for 10 s. PCR primers used are described in Supplementary Table 1.

### Western blotting

After incubation, cells were harvested by scraping and solubilized in 50 mM Tris-HCl (pH 8.0) containing 150 mM NaCl, 1% nonidet P-40, 1 mM PMSF, 1 *μ*g/ml aprotinin, and 1 *μ*g/ml leupeptin. Cells were centrifuged at 1,000 × g for 4 min at 4°C, and pellets were resuspended and stored at -70°C. Extracted proteins (20 *μ*g) were separated by SDS-polyacrylamide gel electrophoresis using 10% polyacrylamide gels, and were electrophoretically transferred onto a PVDF membrane. Membranes were blocked in 5% (w/v) nonfat dried milk in Tris-buffered saline containing 0.1% tween-20 (TBS-T) for 1 h at room temperature. Membranes were then incubated overnight with primary antibodies at a 1:1000 dilution in 5% (w/v) nonfat dried milk in TBS-T at 4°C. Membranes were then incubated with horseradish peroxidase (HRP)-conjugated secondary antibodies. Proteins were visualized by an enhanced chemiluminescence method, and the band intensity was analyzed using a ChemiDoc XRS densitometer and quantified using Quantity One software (Bio-Rad). Protein concentrations were estimated using the bicinchoninic acid method according to the supplier’s recommendations, and bovine serum albumin was used as the standard. Nuclear fractionation was performed using the NE-PER^®^ Nuclear and Cytoplasmic Extraction kit (Thermo Fisher) according to the manufacturer’s protocols.

### Transfection of plasmid DNA and siRNA

Cells were harvested at a density of 1 × 10^6^ cells per 100-mm-dish. Transfection was carried out using the Neon^TM^ transfection system (Invitrogen, Carlsbad, CA). Cells were transfected with 8 *μ*g of plasmid DNA. siGENOME siRNA for human STS (target sequence sense: 5’-CAGUUCAUACAGCGGAACA-3’, antisense: 5’-UGUUCCGCUGUAUGAACU-3’) and siCONTROL nontargeting siRNA (Dharmacon, Lafayette, CO) were used for transfection. Cells were transfected with 50 nM siRNA using the Neon transfection system and cultured in 100-mm dishes in antibiotic-free RPMI with 10 % FBS for 24 h.

### Immunofluorescence

Cells grown on coverslips were washed with PBS and fixed with 3.7% (w/v) paraformaldehyde in 0.1 M sodium phosphate buffer (pH 7.4) for 30 min at room temperature. After washing with PBS, cells were blocked for 15 min in PBS containing 5% goat serum and 0.2 % triton X-100. The cells were then incubated with antibodies (1:500) for 1 h, washed extensively, and stained for 1 h with donkey anti-goat IgG conjugated to Alexa Fluor 594 (Molecular Probe, Eugene, OR). After washing, cells were mounted using Ultra Cruz^TM^ Mounting Medium with DAPI (Santa Cruz) and visualized using a Leica DM 2500 fluorescence microscope (Leica, Wetzlar, Germany).

### STS activity assay

STS activity was measured by incubating cell lysates with DHEAS (50 *μ*M) in 50 mM Tris-HCl (pH 7.4) buffer containing 7 mM MgCl_2_. After 90 min at 37°C, reactions were terminated by the addition of 0.4 ml of chloroform. The products and residual substrate were extracted twice with 0.2 ml of ice-cold chloroform. The organic phase was dried under N_2_ stream, and the residue was dissolved in chloroform and methanol (90:10) for UPLC analysis. The products and residual substrate were separated using a 1.7 *μ*m ACQUITY UPLC BEH C18 column (2.1× 150 mm; Waters, Milford, MA). Mobile phases A and B consisted of ammonium hydroxide (0.02 %, v/v) and methanol/isopropanol (75:25). A gradient program was used at a flow-rate of 0.4 ml/min. The percentage of organic solvent was changed as follows: 0 min, 20% B; 0-7min, 47% B; 7-8 min, 95% B; 8-9 min, 20% B; 9-10 min, 20% B. The absorbance at 450 nm was measured by a Waters ACQUITY PDA detector (Milford, MA).

### Luciferase reporter assay

Cells (1.5 × 10^4^ cells/ well) were cotransfected with 0.4 *μ*g of pcDNA3.1-STS, or TCF/LEF TOPFLASH reporter plasmid vector according to the manufacturer’s protocol using the Neon^TM^ transfection system (Invitrogen). The Twist1 promoter vector was provided by Dr. Mien-Chie Hung (University of Texas, USA). The Twist-HRE mutant promoter vectors were kindly supplied by Dr. H.Y. Lee (Konyang University, Korea). pRL-CMV vector (Promega) was cotransfected as an internal control. After 24 h, the cells were harvested and luciferase activities were measured using the Dual Luciferase Assay System (Promega) with a Synergy^TM^ H1 hybrid microplate reader (Biotek, Winooski, VT).

### Scratch wound assay

Cells (1× 10^6^ cells/well) were cultured in 6-well cell culture plates. After 24 h, cells were washed with PBS and treated with mitomycin C (25 *μ*g/ml) for 30 min. After washing, the confluent monolayer was scratched using sterile pipette tips and then allowed to migrate for 48 h. Plates were photographed using an inverted microscope after the indicated time.

### Cell invasion assay

Cell invasion was measured using a QCM^TM^ 24-well Fluorometric Cell Invasion Assay kit (Millipore), which is composed of 85% type I and 15% type III collagen according to the manufacturer’s instruction. Briefly, cells were seeded onto an invasion chamber insert containing an 8-μm pore size polycarbonate membrane coated with a thin layer of polymerized collagen. Invading cells on the bottom of the insert membrane were visualized and counted under the DM 2500 fluorescence microscope.

### Statistical analysis

Statistical analysis was performed using a one-way analysis of variance and a Dunnett’s pairwise multiple comparison *t*-test using GraphPad Prism (GraphPad Software Inc., San Diego, CA) when appropriate. The difference was considered statistically significant when *p*< 0.05.

## SUPPLEMENTARY MATERIALS TABLE



## References

[1] Saito T (2004). Development of novel steroid sulfatase inhibitors; II. TZS-8478 potently inhibits the growth of breast tumors in postmenopausal breast cancer model rats. J Steroid Biochem Mol Biol.

[2] Purohit A, Foster PA (2012). Steroid sulfatase inhibitors for estrogen- and androgen-dependent cancers. J Endocrinol.

[3] Utsunomiya H (2004). Steroid sulfatase and estrogen sulfotransferase in human endometrial carcinoma. Clin Cancer Res.

[4] Dao TL, Hayes C, Libby PR (1974). Steroid sulfatase activities in human breast tumors. Proc Soc Exp Biol Med.

[5] Nakamura Y (2006). Steroid sulfatase and estrogen sulfotransferase in human prostate cancer. Prostate.

[6] Utsumi T, Yoshimura N, Takeuchi S, Maruta M, Harada N (2000). Elevated steroid sulfatase expression in breast cancers. J Steroid Biochem Mol Biol.

[7] Purohit A, Williams GJ, Howarth NM, Potter BV, Reed MJ (1995). Inactivation of steroid sulfatase by an active site-directed inhibitor, estrone-3-O-sulfamate. Biochemistry.

[8] Arlt W, Haas J, Callies F, Reincke M, Hübler D, Oettel M, Ernst M, Schulte HM, Allolio B (1999). Biotransformation of oral dehydroepiandrosterone in elderly men: significant increase in circulating estrogens. J Clin Endocrinol Metab.

[9] Tworoger SS, Missmer SA, Eliassen AH, Spiegelman D, Folkerd E, Dowsett M, Barbieri RL, Hankinson SE (2006). The association of plasma DHEA and DHEA sulfate with breast cancer risk in predominantly premenopausal women. Cancer Epidemiol Biomarkers Prev.

[10] Hazeldine J, Arlt W, Lord JM (2010). Dehydroepiandrosterone as a regulator of immune cell function. J Steroid Biochem Mol Biol.

[11] Vegliante R, Desideri E, Di Leo L, Ciriolo MR (2016). Dehydroepiandrosterone triggers autophagic cell death in human hepatoma cell line HepG2 via JNK-mediated p62/SQSTM1 expression. Carcinogenesis.

[12] Gilles C, Polette M, Mestdagt M, Nawrocki-Raby B, Ruggeri P, Birembaut P, Foidart JM (2003). Transactivation of vimentin by β-catenin in human breast cancer cells. Cancer Res.

[13] Oviedo PJ, Sobrino A, Laguna-Fernandez A, Novella S, Tarin JJ, Garcia-Perez MA, Sanchis J, Cano A, Hermenegildo C (2011). Estradiol induces endothelial cell migration and proliferation through estrogen receptor-enhanced RhoA/ROCK pathway. Mol Cell Endocrinol.

[14] van Zijl F, Zulehner G, Petz M, Schneller D, Kornauth C, Hau M, Machat G, Grubinger M, Huber H, Mikulits W (2009). Epithelial-mesenchymal transition in hepatocellular carcinoma. Future Oncol.

[15] Anastas JN, Moon RT (2013). WNT signalling pathways as therapeutic targets in cancer. Nat Rev Cancer.

[16] Majid S, Saini S, Dahiya R (2012). Wnt signaling pathways in urological cancers: past decades and still growing. Mol Cancer.

[17] Huber O, Korn R, McLaughlin J, Ohsugi M, Kemier R (1996). Nuclear localization of β-catenin by interaction with transcription factor LEF-1. Mech Dev.

[18] Hedgepeth CM, Deardorff MA, Rankin K, Klein PS (1999). Regulation of glycogen synthase kinase 3β and downstream Wnt signaling by axin. Mol Cell Biol.

[19] Hsu HT, Liu PC, Ku SY, Jung KC, Hong YR, Kao C, Wang C (2006). β-Catenin control of T-cell transcription factor 4 (Tcf4) importation from the cytoplasm to the nucleus contributes to Tcf4-mediated transcription in 293 cells. Biochem Biophys Res Commun.

[20] Luu HH, Zhang R, Haydon RC, Rayburn E, Kang Q, Si W, Park JK, Wang H, Peng Y, Jiang W, He TC (2004). Wnt/β-catenin signaling pathway as a novel cancer drug target. Curr Cancer Drug Targets.

[21] Mann B, Gelos M, Siedow A, Hanski ML, Gratchev A, Ilyas M, Bodmer WF, Moyer MP, Riecken EO, Buhr HJ, Hanski C (1999). Target genes of β-catenin-T cell-factor/lymphoid-enhancer-factor signaling in human colorectal carcinomas. Proc Natl Acad Sci U S A.

[22] Day TF, Guo X, Garrett-Beal L, Yang Y (2005). Wnt/β-catenin signaling in mesenchymal progenitors controls osteoblast and chondrocyte differentiation during vertebrate skeletogenesis. Dev Cell.

[23] Sanchez-Tillo E, de Barrios O, Siles L, Cuatrecasas M, Castells A, Postigo A (2011). β-catenin/TCF4 complex induces the epithelial-to-mesenchymal transition (EMT)-activator ZEB1 to regulate tumor invasiveness. Proc Natl Acad Sci U S A.

[24] Liu C, Li Y, Semenov M, Han C, Baeg GH, Tan Y, Zhang Z, Lin X, He X (2002). Control of β-catenin phosphorylation/degradation by a dual-kinase mechanism. Cell.

[25] Cross DA, Alessi DR, Cohen P, Andjelkovich M, Hemmings BA (1995). Inhibition of glycogen synthase kinase-3 by insulin mediated by protein kinase B. Nature.

[26] Novak A, Hsu SC, Leung-Hagesteijn C, Radeva G, Papkoff J, Montesano R, Roskelley C, Grosschedl R, Dedhar S (1998). Cell adhesion and the integrin-linked kinase regulate the LEF-1 and β-catenin signaling pathways. Proc Natl Acad Sci U S A.

[27] Hayashida Y, Honda K, Idogawa M, Ino Y, Ono M, Tsuchida A, Aoki T, Hirohashi S, Yamada T (2005). E-cadherin regulates the association between β-catenin and actinin-4. Cancer Res.

[28] Kim AY, Kwak JH, Je NK, Lee YH, Jung YS (2015). Epithelial-mesenchymal transition is associated with acquired resistance to 5-fluorocuracil in HT-29 colon cancer cells. Toxicol Res.

[29] Eldar-Finkelman H, Seger R, Vandenheede JR, Krebs EG (1995). Inactivation of glycogen synthase kinase-3 by epidermal growth factor is mediated by mitogen-activated protein kinase/p90 ribosomal protein S6 kinase signaling pathway in NIH/3T3 cells. J Biol Chem.

[30] McManus EJ, Sakamoto K, Armit LJ, Ronaldson L, Shpiro N, Marquez R, Alessi DR (2005). Role that phosphorylation of GSK3 plays in insulin and Wnt signalling defined by knockin analysis. EMBO J.

[31] Shin SY, Yoon SC, Kim YH, Kim YS, Lee YH (2002). Phosphorylation of glycogen synthase kinase-3β at serine-9 by phospholipase Cγ1 through protein kinase C in rat 3Y1 fibroblasts. Exp Mol Med.

[32] Gradl D, Kuhl M, Wedlich D (1999). The Wnt/Wg signal transducer β-catenin controls fibronectin expression. Mol Cell Biol.

[33] Yang J, Mani SA, Donaher JL, Ramaswamy S, Itzykson RA, Come C, Savagner P, Gitelman I, Richardson A, Weinberg RA (2004). Twist, a master regulator of morphogenesis, plays an essential role in tumor metastasis. Cell.

[34] Planas-Silva MD, Waltz PK (2007). Estrogen promotes reversible epithelial-to-mesenchymal-like transition and collective motility in MCF-7 breast cancer cells. J Steroid Biochem Mol Biol.

[35] Casas E, Kim J, Bendesky A, Ohno-Machado L, Wolfe CJ, Yang J (2011). Snail2 is an essential mediator of Twist1-induced epithelial mesenchymal transition and metastasis. Cancer Res.

[36] Cho KH, Jeong KJ, Shin SC, Kang J, Park CG, Lee HY (2013). STAT3 mediates TGF-β1-induced TWIST1 expression and prostate cancer invasion. Cancer Lett.

[37] Puisieux A, Brabletz T, Caramel J (2014). Oncogenic roles of EMT-inducing transcription factors. Nat Cell Biol.

[38] Suh BY, Jung JJ, Park N, Seong CH, Im HJ, Kwon Y, Kim D, Chun YJ (2011). Induction of steroid sulfatase expression by tumor necrosis factor-α through phosphatidylinositol 3-kinase/Akt signaling pathway in PC-3 human prostate cancer cells. Exp Mol Med.

[39] Sung CH, Im HJ, Park N, Kwon Y, Shin S, Ye DJ, Cho NH, Park YS, Choi HK, Kim D, Chun YJ (2013). Induction of steroid sulfatase expression in PC-3 human prostate cancer cells by insulin-like growth factor II. Toxicol Lett.

[40] Kulendran M, Salhab M, Mokbel K (2009). Oestrogen-synthesising enzymes and breast cancer. Anticancer Res.

[41] Orsulic S, Huber O, Aberle H, Arnold S, Kemler R (1999). E-cadherin binding prevents β-catenin nuclear localization and β-catenin/LEF-1-mediated transactivation. J Cell Sci.

[42] Conacci-Sorrell M, Simcha I, Ben-Yedidia T, Blechman J, Savagner P, Ben-Ze'ev A (2003). Autoregulation of E-cadherin expression by cadherin-cadherin interactions: the roles of β-catenin signaling, Slug, and MAPK. J Cell Biol.

[43] Liu X, Arnold JT, Blackman MR (2010). Dehydroepiandrosterone administration or Gαq overexpression induces β-catenin/T-Cell factor signaling and growth via increasing association of estrogen receptor-β/Dishevelled2 in androgen-independent prostate cancer cells. Endocrinology.

[44] MacDonald BT, Tamai K, He X (2009). Wnt/β-catenin signaling: components, mechanisms, and diseases. Dev Cell.

[45] Cheng GZ, Chan J, Wang Q, Zhang W, Sun CD, Wang LH (2007). Twist transcriptionally up-regulates AKT2 in breast cancer cells leading to increased migration, invasion, and resistance to paclitaxel. Cancer Res.

[46] Li J, Zhou BP (2011). Activation of β-catenin and Akt pathways by Twist are critical for the maintenance of EMT associated cancer stem cell-like characters. BMC Cancer.

[47] Fusi L, Purohit A, Brosens J, Woo LW, Potter BV, Reed MJ (2008). Inhibition of steroid sulfatase activity in endometriotic implants by STX64 (667Coumate): a potential new therapy. ScientificWorldJournal.

[48] Woo LW, Ganeshapillai D, Thomas MP, Sutcliffe OB, Malini B, Mahon MF, Purohit A, Potter BV (2011). Structure-activity relationship for the first-in-class clinical steroid sulfatase inhibitor Irosustat (STX64, BN83495). ChemMedChem.

[49] Batlle E, Sancho E, Francí C, Domínguez D, Monfar M, Baulida J, García De Herreros A (2000). The transcription factor snail is a repressor of E-cadherin gene expression in epithelial tumour cells. Nat Cell Biol.

[50] Eckert MA, Lwin TM, Chang AT, Kim J, Danis E, Ohno-Machado L, Yang J (2011). Twist1-induced invadopodia formation promotes tumor metastasis. Cancer Cell.

[51] Ansieau S, Bastid J, Doreau A, Morel AP, Bouchet BP, Thomas C, Fauvet F, Puisieux I, Doglioni C, Piccinin S, Maestro R, Voeltzel T, Selmi A (2008). Induction of EMT by twist proteins as a collateral effect of tumor-promoting inactivation of premature senescence. Cancer Cell.

[52] Yuen HF, Chua CW, Chan YP, Wong YC, Wang X, Chan KW (2007). Significance of TWIST and E-cadherin expression in the metastatic progression of prostatic cancer. Histopathology.

[53] Yang MH, Wu MZ, Chiou SH, Chen PM, Chang SY, Liu CJ, Teng SC, Wu KJ (2008). Direct regulation of TWIST by HIF-1α promotes metastasis. Nat Cell Biol.

[54] Xu Y, Li Y, Pang Y, Ling M, Shen L, Yang X, Zhang J, Zhou J, Wang X, Liu Q (2012). EMT and stem cell-like properties associated with HIF-2α are involved in arsenite-induced transformation of human bronchial epithelial cells. PLoS One.

[55] Carroll VA, Ashcroft M (2006). Role of hypoxia-inducible factor (HIF)-1α versus HIF-2α in the regulation of HIF target genes in response to hypoxia, insulin-like growth factor-I, or loss of von Hippel-Lindau function: implications for targeting the HIF pathway. Cancer Res.

[56] Gort EH, van Haaften G, Verlaan I, Groot AJ, Plasterk RH, Shvarts A, Suijkerbuijk KP, van Laar T, van der Wall E, Raman V, van Diest PJ, Tijsterman M, Vooijs M (2008). The TWIST1 oncogene is a direct target of hypoxia-inducible factor-2α. Oncogene.

[57] Woo LL, Purohit A, Malini B, Reed MJ, Potter BV (2000). Potent active site-directed inhibition of steroid sulphatase by tricyclic coumarin-based sulphamates. Chem Biol.

[58] Ye DJ, Kwon YJ, Shin S, Baek HS, Shin DW, Chun YJ (2017). Induction of integrin signaling by steroid sulfatase in human cervical cancer cells. Biomol Ther.

[59] Adiguzel E, Hou G, Sabatini PJ, Bendeck MP (2013). Type VIII collagen signals via β1 integrin and RhoA to regulate MMP-2 expression and smooth muscle cell migration. Matrix Biol.

[60] Morozevich G, Kozlova N, Cheglakov I, Ushakova N, Berman A (2009). Integrin α5β1 controls invasion of human breast carcinoma cells by direct and indirect modulation of MMP-2 collagenase activity. Cell Cycle.

[61] Yang J, Hou Y, Zhou M, Wen S, Zhou J, Xu L, Tang X, Du YE, Hu P, Liu M (2016). Twist induces epithelial-mesenchymal transition and cell motility in breast cancer via ITGB1-FAK/ILK signaling axis and its associated downstream network. Int J Biochem Cell Biol.

