# Supplementary material for: Human steroid sulfatase induces Wnt/β-catenin signaling and epithelial-mesenchymal transition by upregulating Twist1 and HIF-1α in human prostate and cervical cancer cells
Source: Oncotarget. 2017 Jun 27;8(37):61604–17. doi: 10.18632/oncotarget.18645 (PMC5617449; doi:10.18632/oncotarget.18645)
Supplement: Supplementary file 1 [file oncotarget-08-61604-s001.pdf]

Human steroid sulfatase induces Wnt/β-catenin signaling and epithelial-mesenchymal transition by upregulating Twist1 and HIF-1α in human prostate and cervical cancer cells

SUPPLEMENTARY MATERIAL

Supplementary Table 1: DNA sequences of PCR primers used in this study

**STS**	Sense	5'-CCTCCTACTGTTCTTTCTGTGGG-3’
	Antisense	5'-GGTCGATATTGGGAGTCCTGATA-3’
**c-Myc**	Sense	5'-GGCTCCTGGCAAAAGGTCA-3’
	Antisense	5'-AGTTGTGCTGATGTGTGGAGA-3’
**Cyclin D1**	Sense	5'-ACCTGAGGAGCCCCAACA-3’
	Antisense	5'-TCTGCTCCTGGCAGGCC-3’
**Zeb1**	Sense	5’-TCCTGAGGCACCTGAAGAGG-3’
	Antisense	5’-CAGAGAGGTAAAGCGTTTATAGCC-3’
**Zeb2**	Sense	5'-GGAGACGAGTCCAGCTAGTGT-3’
	Antisense	5'-CCACTCCACCCTCCCTTATTTC-3’
**Snail**	Sense	5'-TCGGAAGCCTAACTACAGCGA-3’
	Antisense	5'-AGATGAGCATTGGCAGCGAG-3’
**HIF-1α**	Sense	5’-GAAAGCGCAAGTCTTCAAAG-3’
	Antisense	5’-TGGGTAGGAGATGGAGATGC-3’
**FOXC2**	Sense	5'-CCTCCTGGTATCTCAACCACA-3’
	Antisense	5'-GAGGGTCGAGTTCTCAATCCC-3’
**Twist1**	Sense	5'-GGGAGTCCGCAGTCTTAC-3’
	Antisense	5'-CCTGTCTCGCTTTCTCTTT-3’
**E-cadherin**	Sense	5'-AAAGGCCCATTTCCTAAAAACCT-3’
	Antisense	5'-TGCGTTCTCTATCCAGAGGCT'-3’
**N-cadherin**	Sense	5'- CACCCAACATGTTTACAATCAACAATGAGAC-3’
	Antisense	5'- CTGCAGCAACAGTAAGGACAAACATCCTATT-3’
**MMP-1**	Sense	5'-TGGACCATGCCATTGAGAAA-3’
	Antisense	5'-CCGATCATCTCCCCTGACAA'-3’
**MMP-2**	Sense	5'- TGATCTTGACCAGAATACCATCGA -3’
	Antisense	5'-GGCTTGCGAGGGAAGAAGTT-3’
**MMP-7**	Sense	5'-GTGGTCACCTACAGGATCGTA-3’
	Antisense	5'-CTGAAGTTTCTATTTCTTTCTTGA-3’
**MMP-9**	Sense	5'-GTGCTGGGCTGCTGCTTTGCT -3’
	Antisense	5’- GTCGCCCTCAAAGGTTTGGAA -3’
